# Statistical analysis of multiple regions-of-interest in multiplexed spatial proteomics data

**DOI:** 10.1093/bib/bbae522

**Published:** 2024-10-20

**Authors:** Sarah Samorodnitsky, Michael C Wu

**Affiliations:** Public Health Sciences Division, Fred Hutchinson Cancer Center, Seattle, WA 98109, United States; SWOG Statistics and Data Management Center, Fred Hutchinson Cancer Center, Seattle, WA 98109, United States; Public Health Sciences Division, Fred Hutchinson Cancer Center, Seattle, WA 98109, United States; SWOG Statistics and Data Management Center, Fred Hutchinson Cancer Center, Seattle, WA 98109, United States

**Keywords:** multiplexed spatial proteomics, spatial point process, regions-of-interest, multiplexed immunofluorescence, single-cell data

## Abstract

Multiplexed spatial proteomics reveals the spatial organization of cells in tumors, which is associated with important clinical outcomes such as survival and treatment response. This spatial organization is often summarized using spatial summary statistics, including Ripley’s K and Besag’s L. However, if multiple regions of the same tumor are imaged, it is unclear how to synthesize the relationship with a single patient-level endpoint. We evaluate extant approaches for accommodating multiple images within the context of associating summary statistics with outcomes. First, we consider averaging-based approaches wherein multiple summaries for a single sample are combined in a weighted mean. We then propose a novel class of ensemble testing approaches in which we simulate random weights used to aggregate summaries, test for an association with outcomes, and combine the $P$-values. We systematically evaluate the performance of these approaches via simulation and application to data from non-small cell lung cancer, colorectal cancer, and triple negative breast cancer. We find that the optimal strategy varies, but a simple weighted average of the summary statistics based on the number of cells in each image often offers the highest power and controls type I error effectively. When the size of the imaged regions varies, incorporating this variation into the weighted aggregation may yield additional power in cases where the varying size is informative. Ensemble testing (but not resampling) offered high power and type I error control across conditions in our simulated data sets.

## Introduction

The spatial architecture of immune cells in the tumor microenvironment (TME) is an important driver of patient-level outcomes in oncology, such as survival [1–4] and treatment response [5, 6]. This architecture can be revealed by multiplexed spatial proteomics imaging platforms, such as codetection by imaging (CODEX) [7] and multiplexed ion beam imaging [8]. These platforms generate high-resolution images illustrating the spatial expression of proteins which, in turn, can be used to derive the phenotype (e.g. CD8 T cell) and function (e.g. initiating an immune response) of cells residing within the TME [9]. Spatially-resolved imaging of the TME allows investigators to study how cellular organization within and around tumors influences patient-level outcomes in the clinic.

**Figure 1:**
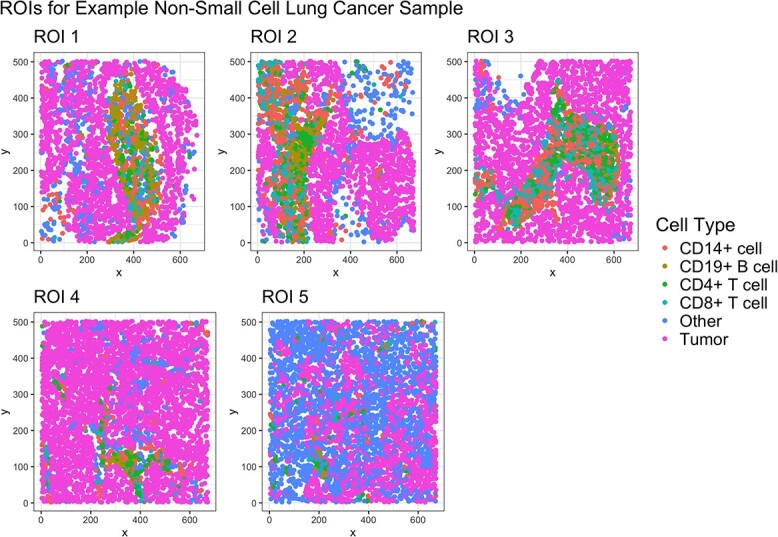
Five ROIs from a non-small cell lung cancer tumor biopsy. Each ROI shows a unique pattern of immune (CD14+ cells, CD19+ B cells, CD4+ T cells, CD8+ T cells), tumor, and other cell types.

To comprehensively characterize the composition of the TME, multiple regions-of-interest (ROIs) may be imaged from the same tumor biopsy (Fig. 1) [10]. These ROIs, however, may not be adjacent so they cannot be treated as a single image [9]. To handle multiple ROIs in an associative analysis with clinical outcomes, one solution is to select the ROI from each patient with the most cells (or any other characteristic) [11]. An alternative is to use a spatial summary statistic, like Ripley’s K [12], Besag’s L [13], or the g-function [14], to quantify the spatial distribution of cells in each ROI and then compute an average within each individual [15]. Unfortunately, selecting an image that does not well characterize the association with outcomes or using a sub-optimal set of weights in constructing the average leads to reduced power. Following more standard spatial analysis literature [16, 17], others have used random effects (e.g., within a linear mixed model) for multiplexed imaging [18, 19]. These studies treat a summary statistic for each image as the outcome variable. However, these methods can be difficult to generalize to more sophisticated clinical outcomes: it is unclear how to use censored survival data as a predictor. Reversing the models to keep the clinical outcome as the dependent variable requires use of specialized random effects models for each type of outcome and, more problematically, may have difficulties in controlling type I error in practice, e.g. due to incorrect specification of mixing distributions.

The main objective of this article is to compare approaches for aggregating multiple spatial summary statistics into a single value that is used in testing for an association with clinical outcomes. The goal is to describe whether there is an association between the spatial distribution of cells across ROIs with patient-level outcomes. We consider the power and validity (false positive control) of testing for this association using three different weighted averages of spatial summaries. In addition, given that the optimal (most powerful) set of weights is unknown *a priori*, we further consider three new ensemble testing approaches that hedge against different scenarios and work well in the omnibus. These approaches simulate random weights used to construct an average summary for each sample, test for an association with a sample-level outcome, and combine the $P$-values across many ensemble replications. We narrow our scope to hypothesis testing, given a survival and a binary outcome as clinical outcomes are often in these forms, such as case vs. control status or overall survival. We examine the performance of these methods via simulation studies and also consider three multiplexed spatial proteomics data applications in non-small cell lung cancer, colorectal cancer (CRC), and triple negative breast cancer (Supplementary Materials).

## Methods

In this section, we first describe our notation and review existing spatial summary metrics that are commonly used. We then discuss possible aggregation and averaging approaches before presenting additional proposed ensemble approaches.

Consider a dataset with $N$ samples. For each sample, we have $R_{i}$, $i=1,\dots , N$ single cell images of different ROIs from a tissue biopsy, e.g. tumor sample. Let $n_{ir}$ represent the number of detected cells in image $r$ and $A_{ir}$ represent the area of image $r$, where $r=1, \dots , R_{i}$. The analytic objective is to determine whether global spatial characteristics of the images are associated with a generally specified outcome $y$.

### Review of spatial summary statistics

Many multiplexed imaging methods rely on the spatial point process model to describe the spatial distribution of cells in tissue [9]. The spatial point process model is a probability model that gives rise to the random arrangement of points in space, often in 2D or 3D. A realization of a spatial point process is termed a point pattern, i.e. the distribution of cells observed in a multiplexed spatial proteomics image [14]. The point pattern may be ‘marked’, meaning we possess attributes associated with each point or, in our case, cell [14]. These could be categorical cell phenotypes, e.g. CD8 T cell, or continuous marker expression, e.g. the expression level of the CD8 protein. We focus here on categorical marks as it is often of interest to relate the spatial distribution of specific cell types to patient outcomes. A common spatial point process model (marked or unmarked) is the homogeneous Poisson point process. This model is characterized by exhibiting a homogeneous intensity, meaning the expected number of cells per unit area is the uniform across locations, and exhibiting complete spatial randomness or CSR [14]. CSR implies that the regions within the observed point pattern are independent of each other and the point locations may be anywhere within the image.

It is often of interest to test whether a point pattern arises from a point process exhibiting CSR. Deviations from this hypothesis imply that the pattern exhibits clustering (cells are more clustered together than expected by random chance) or dispersion (cells are further apart than expected by random chance). To test whether the whether point process exhibits CSR, it is common to use spatial summary statistics, such as Ripley’s K [12] or Besag’s L [13], which quantify the degree of adherence or deviation to CSR at a specific radius $t$. For a point pattern $X$ in ROI $r$ of sample $i$, Ripley’s K function is defined as follows:


(1) \begin{align*}& K(t) = \frac{1}{\lambda}\mathbb{E}\left(\#\ \text{of points within distance } t \text{ of } u \mid X \text{ has a point at } u\right)\end{align*}

where $\lambda $ is the intensity of the image. Under CSR, the empirical Ripley’s K for a single cell type $a$ is defined as follows:


(2) \begin{align*}& K^{a}_{ir}(t) = \frac{1}{\lambda^{a}_{ir}} \sum_{j=1}^{n_{ir}} \sum_{j^{\prime} \neq j} w^{-1}_{jj^{\prime}} \frac{1(d_{jj^{\prime}} \leq t)}{n^{a}_{ir}}\end{align*}

where $\lambda ^{a}_{ir} = n^{a}_{ir}/A_{ir}$ is the estimated intensity of ROI $r$ and $d_{jj^{\prime}}$ is the distance between the $j$th and $j^{\prime}$th cells of type $a$. $w_{jj^{\prime}}$ is an edge correction used to adjust the contribution of $1(d_{jj^{\prime}} \leq t)$ when the radius $t$ crosses the edge of the image. The value of $K(t)$ implies whether the point pattern exhibits CSR: if the value of $K^{a}_{ir}(t)$ across a range of $t$ is approximately equal to $\pi t^{2}$ then we conclude affirmatively. If $K^{a}_{ir}(t)> \pi t^{2}$, then we conclude it exhibits clustering, and if $K^{a}_{ir}(t) < \pi t^{2}$, then it exhibits dispersion. Ripley’s K can be generalized to describe the bivariate colocalization patterns between two cell types, $a$ and $b$:


(3) \begin{align*}& \hat K^{ab}_{ir}(t) = \left(\hat\lambda_{ir}^{a} \hat\lambda_{ir}^{b} A_{ir} \right)^{-1} \sum_{j}^{n^{a}_{ir}} \sum_{j^{\prime}}^{n^{b}_{ir}} w^{-1}_{a_{j} b_{j^{\prime}}} I(d_{a_{j}, b_{j^{\prime}}} < t)\end{align*}

Besag’s L is a transformed version of Ripley’s K: $L(t) = \sqrt{K(t)/\pi }$ for both univariate and bivariate scenarios. It has a similar interpretation as $K(t)$: if $L(t) \approx t$, the point process exhibits CSR, if $L(t)> t$, it exhibits clustering, and if $L(t) < t$, it exhibits dispersion. In addition to testing CSR, $K(t)$ and $L(t)$ may be used to describe the spatial distribution of cells at a radius $t$. This has been shown to be clinically meaningful in several applications [15, 19, 20].

When using Ripley’s K and Besag’s L descriptively, these statistics may be treated as covariates in an outcome model. A challenge arises when multiple ROIs per tissue sample are collected. In this case, we can compute multiple estimates of the spatial summary for the same sample. To accommodate multiple summaries and relate them to a single outcome measurement, we can aggregate the summaries into a single value. We now review three aggregation recipes to combine spatial summary statistics.

### Aggregation methods for multiple spatial summary statistics

It is common in spatial proteomics imaging studies for multiple regions of a tissue to be imaged. Obtaining multiple images per sample yields multiple spatial summary statistics characterizing the level of clustering or dispersion within each image. When using spatial summary statistics, like Ripley’s K, to describe the spatial distribution of cells in each ROI, one must decide how to associate the summaries to an outcome when there is a single endpoint per sample. A straightforward option is to aggregate the spatial summaries within a sample into a single value using a weighted average.

Diggle *et al*. (1991) [21], Baddeley *et al*. (1993) [22], and Landau *et al*. (2004) [23] each proposed an approach to aggregating multiple Ripley’s K summaries:


(4) \begin{align*} \hat K_{i}(t) = \frac{1}{\sum_{r=1}^{R_{i}} n_{ir}} \sum_{r=1}^{R_{i}} n_{ir} \hat K_{ir}(t) && \text{Diggle}\ \textit{et\, al}.\ \text{(1991)} \end{align*}

(5) \begin{align*} \hat K_{i}(t) = \frac{1}{\sum_{r=1}^{R_{i}} n_{ir}^{2}} \sum_{r=1}^{R_{i}} n_{ir}^{2} \hat K_{ir}(t) && \text{Baddeley}\ \textit{et\, al}.\ \text{(1993)} \end{align*}

(6) \begin{align*} \hat K_{i}(t) = \frac{\sum_{r=1}^{R_{i}} A_{ir}}{\left( \sum_{r=1}^{R_{i}} n_{ir} \right)^{2}} \sum_{r=1}^{R_{i}} \frac{n_{ir}^{2}}{A_{ir}} \hat K_{ir}(t) && \text{Landau }\,\textit{et\, al}.\ \text{(2004)}\end{align*}

The motivation behind these aggregation recipes is to weight the Ripley’s K statistics by their sampling variances. The sampling variance of Ripley’s K, however, is difficult to write down explicitly, so the weights are chosen to be proportional to this expression. When the intensities of each image are assumed to be the same (i.e., $\lambda _{ir} = n_{ir}/A_{ir} \approx n_{ir^{\prime}}/A_{ir^{\prime}} = \lambda _{ir^{\prime}}$, where $r\neq r^{\prime}$), Equations 4, 5, and 6 are equivalent. Conditional on the $n_{ir}$s and assuming the sampling variances of $\hat K_{ir}(t)$ are proportional to $n_{ir}^{-2}$, Equation 5 was shown to be the best linear unbiased estimator of $K_{i}(t)$ [22, 23]. This is a reasonable assumption if the image areas are the same [24]. If the intensities differ across images, then the sampling variance of $\hat K_{ir}(t)$ is proportional to $\lambda _{ir}^{-1} n_{ir}^{-1}$ [24]. In this case, Equation 6 should account for the variation in intensity, as $n_{ir}/A_{ir}$ is an estimate of the intensity in ROI $r$ of image $i$.

The aggregations given in Equations 4, 5, and 6 were originally described to combine estimates of univariate Ripley’s K derived from replicated spatial point patterns. Replicated point patterns arise from repeated observations of the same experiment [14] which in our case could be thought of as imaging the same tissue region multiple times. In our context, the ROIs do not necessarily reflect replicated spatial point patterns since they arise from different locations within the same sample. Despite that, they may exhibit spatial correlation, which motivates our use of these aggregation methods. Moreover, it is often of interest to describe the spatial colocalization of two cell types in spatial proteomics imaging. We consider applying these aggregation methods to multiple bivariate Ripley’s K statistics and describe the operating characteristics thereof. For clarity, we treat the total number of cells and the total image area as the weights in the above weighted aggregations.

### Ensemble testing using randomly-weighed averages

Weighted aggregation methods are a straightforward avenue for combining multiple estimates of a spatial summary statistic. However, they were largely developed with the understanding that multiple images were simply repeated measures and assume some degree of homogeneity. When images are very disparate, averaging may be less desirable and may reduce power due to relying on an inadequate set of weights to combine spatial summary estimates. For these situations, we also consider the use of ensemble approaches for testing associations with clinical outcomes.

Ensemble approaches for prediction have grown in popularity and demonstrated impressive performance [25]. Liu *et al*. (2023) [26] recently brought the idea of ensemble prediction to hypothesis testing. The idea of ensemble testing is to combine multiple weak test statistics to form a more powerful test for a global null of the form $H_{0}: \boldsymbol{\beta } = 0$, where $\boldsymbol{\beta } = (\beta _{1}, \dots , \beta _{P})^{T}$. The authors proposed simulating random weights to combine the score test statistics used to test each marginal hypothesis, $H_{0}: \beta _{p}=0$ for $p=1,\dots , P$, into a single global test. They suggested to repeat this process many times and combine the resulting $P$-values using the Cauchy combination test [27]. We adapt this idea to our context by generating random weights used to construct an aggregation of multiple spatial summary statistics across ROIs. We repeat this process multiple times, each time testing for an association between each random aggregation and clinical outcomes. At the end, we combine the resulting $P$-values using the Cauchy combination test to yield a global, omnibus test of association.

We consider three variations of ensemble testing in our study. We refer to our first approach as the general ‘ensemble’ test as it most closely mirrors the framework used in Liu *et al*. (2023). For each person with $R_{i}$ ROIs, we randomly sample a vector of weights $\boldsymbol{\omega } = (\omega _{1}, \dots , \omega _{R_{i}})$ iid from a standard normal distribution, $N(0,1)$. We take the absolute value of each weight and scale the weights to sum to one, i.e. $\boldsymbol{\omega }^{*} = (\omega _{1}^{*}, \dots , \omega _{R_{i}}^{*}) = (|\omega _{1}|/||\boldsymbol{\omega }||, \dots , |\omega _{R_{i}}|/||\boldsymbol{\omega }||)$. We then compute a weighted aggregation based on this random sequence of weights for each person: $\hat K^{ensemble}_{i}(t) = \frac{1}{\sum _{r=1}^{R_{i}} \omega ^{*}_{r}} \sum _{r=1}^{R_{i}} \omega ^{*}_{r} \hat K_{ir}(t)$. We perform this procedure for each sample to yield a single aggregated spatial summary statistic for each sample $i$. Given $\boldsymbol{\omega }^{*}$, we test for an association between $\hat K^{ensemble}_{i}(t), i=1,\dots , N$ with the outcome and store the resulting $P$-value. We repeat this procedure $B$ times (e.g. 1000 times) to generate $B$$P$-values which are then combined using a truncated Cauchy combination test. To truncate the $P$-values, we round all $P$-values greater than $0.5$ down to the nearest $0.1$ to increase power. We denote these truncated $P$-values as $p^{*}_{i}$. Then, the Cauchy combination test statistic is


(7) \begin{align*}& T_{omni} = \sum_{i=1}^{B} \alpha_{i} \tan\left[ \pi (0.5 - p^{*}_{i})\right]\end{align*}

Under the null, $T_{omni}\sim \hbox{Cauchy}(0,1)$, which we use to calculate an omnibus $P$-value. For simplicity, we fix the weights $\alpha _{i} = 1/B$.

We also consider a ‘resampling’ approach in which we randomly select one spatial summary statistic to represent each sample. Effectively, we perform the process described in the ‘ensemble’ approach but fix the weights at $1$ or $0$ and require exactly one $\omega _{r} = 1$ and $\omega _{r^{\prime}}=0$ for $r\neq r^{\prime}$ and $r, r^{\prime} = 1,\dots , R_{i}$. We repeat this process many times, e.g. 1000 times. Within each replication, we test for an association between the vector of $\hat K^{resample}_{i}(t)$ for each sample and the outcome-of-interest. The resulting $P$-values are aggregated using the truncated Cauchy combination test described above.

Finally, we consider a blend of the Diggle aggregator given in Equation 4 and the ‘ensemble’ approach. We refer to this as the ‘combination’ or ‘combo’ test. As with the general ensemble test, we randomly generate a set of weights, $\boldsymbol{\omega } = (\omega _{1}, \dots , \omega _{R_{i}})$, for the images within each sample from a standard normal distribution and scale the weights to sum to one, yielding $\boldsymbol{\omega }^{*} = (\omega ^{*}_{1}, \dots , \omega ^{*}_{R_{i}})$. In addition, we add a weight for the number of cells in each image. Our final aggregator is then $\hat K^{combo}_{i}(t) = \frac{1}{\sum _{r=1}^{R_{i}} \omega ^{*}_{r} n_{ir}} \sum _{r=1}^{R_{i}} \omega ^{*}_{r} n_{ir} \hat K_{ir}(t)$. As before, for each replication, we test for an association between $\hat K^{combo}_{i}(t), i=1,\dots , N$ and the outcome-of-interest and aggregate the resulting $P$-values using the truncated Cauchy combination test.

### Simulation study

We use simulation studies to compare the power and type I error rates of each of the aggregation methods described above: the Diggle *et al*. (1991) mean (Equation 4), the Baddeley *et al*. (1993) mean (Equation 5), the Landau *et al*. (2004) mean (Equation 6), the ensemble approach, the resampling approach, and the combo approach. In addition, we consider a standard arithmetic mean of the spatial summary statistics, henceforth referred to as the ‘mean’ approach. While not a recommended strategy, we also considered treating each image as independent, ignoring repeated measures among the samples, and test for an association between the images and the sample-level outcome. We refer to this method as the ‘no aggregation’ approach.

In our simulation study, we varied the spatial distribution of cells as represented by a spatial summary statistic, the area of each image, and the number of cells per image. To generate realistic spatial summary statistics, image areas, and numbers of points, we based our simulation study on a real dataset from a study of non-small cell lung cancer (NSCLC) [28]. In this study of $153$ participants, multiple regions of lung adenocarcinoma tumor biopsies were imaged using multiplexed immunohistochemistry. This yielded between four and six images per person for a total of $761$ images across participants. To generate data for our simulation study, we estimated Besag’s L at a radius of $10$ for each image using the tumor cells detected in the tumor compartment of each sample. The spatial summaries were highly right-skewed so we used a $\log (1+x)$ transformation to normalize the values. We removed three images in which there were zero tumor cells or only one detected tumor cell. This yielded $758$ spatial summaries to use in our simulation study. We also retained the area and number of tumor cells in each of these $758$ images.

To simulate spatial summaries, we fixed the total sample size of our simulation study at $n=153$ to mimic the original NSCLC study. For each sample, we randomly chose to simulate between 5 and 10 images. We then generated spatial summary statistics, image areas, and numbers of cells independently from an interpolated empirical cumulative distribution function (eCDF) for each of these three parameters. The interpolated eCDF was constructed based on values unique to each sample. This was done to yield spatial summaries similar to what was truly observed with some random variation. For each sample and simulation replication, we simulated spatial summaries denoted $L^{*}_{ir}$, $ n^{*}_{ir}$, and $ A^{*}_{ir}$, where $i$ indexes the sample and $r$ indexes the ROI within that sample.

We considered survival as the sample-level outcome as it is often used as the clinical endpoint in TME studies in cancer. We simulated survival times from an exponential distribution via


(8) \begin{align*}& S^{*}_{i} = -\frac{\log(U_{i})}{\exp(1 + \beta \bar L^{*}_{i})}\end{align*}

where $U_{i} \sim \hbox{Uniform}(0,1)$, $\beta $ is an effect size that was varied throughout the study and $\bar L^{*}_{i}$ represents the mean of the simulated spatial summaries for sample $i$. We varied the mean aggregation used to compute $\bar L^{*}_{i}$: an arithmetic mean, the Diggle mean, the Baddeley mean, and the Landau mean. We varied the effect size across $\beta = -3, -2.5, -2, -1.5, -1$. We report the power for $1,000$ simulation replications and type I error rates for $10\,000$ simulation replications of the seven aggregation methods and treating each ROI independently (‘no aggregation’). For the ensemble approaches, we use $1000$ ensemble replications.

## Results

### Simulation study

**Figure 2:**
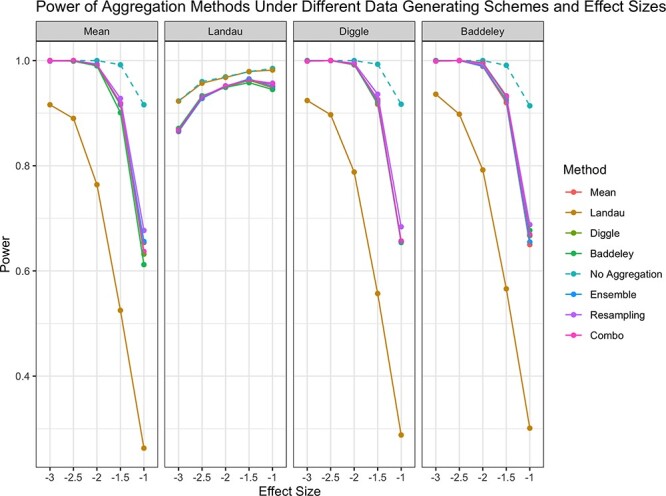
Power of each aggregation method in testing for an association between spatial summaries across images and survival. Each panel illustrates results for a different ‘true’ aggregator associated with the outcome. ‘No Aggregation’ refers to treating each spatial summary independently within a sample. Line corresponding to ‘No Aggregation’ is dashed to indicate that this is not a recommended approach to handling multiple spatial summaries.

The power of each aggregation method is shown in Fig. 2. We found that across a range of true aggregators (the aggregation method associated with survival) and effect sizes, the arithmetic mean, the Diggle mean, the Baddeley mean, ensemble testing, resampling, and the combo approach all provided the highest power. Only when the Landau mean was the true aggregator did it outperform the other approaches. This suggests the Landau mean performs best when the intensity of the images is informative in aggregating the spatial summary statistics. This may be the case in settings where the intensity varies across ROIs within a sample.

**Figure 3:**
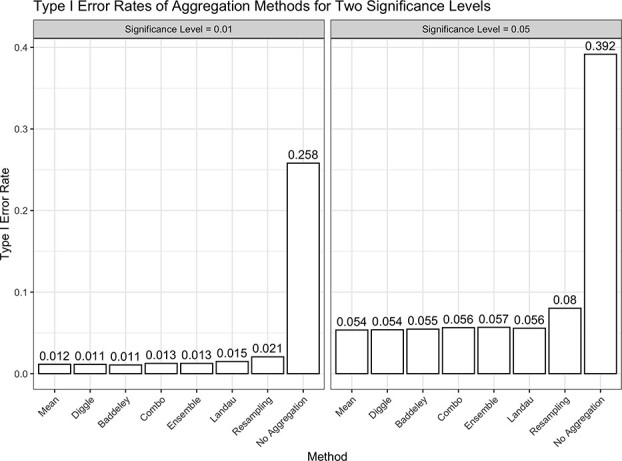
Type I error rates of each aggregation method in testing for an association between spatial summaries across images and survival. ‘No Aggregation’ refers to treating each spatial summary independently within a sample. As illustrated, it is not a reasonable approach to handling multiple spatial summary statistics because of its high type I error rate.

The type I error rate of each aggregation method under simulation is shown in Fig. 3 for a significance level of $0.01$ and $0.05$. At both levels, an arithmetic mean, the Diggle mean, the Baddeley mean, the Landau mean, the ensemble approach, and the combination approach controlled the type I error rate at approximately the nominal level. Surprisingly, at both levels, resampling exhibited slightly elevated type I errors around 2% for the 0.01 level and 8% for the 0.05 level, suggesting it may not be a valid hypothesis test. As expected, treating each image independently resulted in an excessively high type I error.

We also compared the ensemble approaches based on their computational speed. For 1000 ensemble replications, on average, the ensemble method took 3.2 seconds, the resampling method took 6.1 seconds, and the combination approach took 3.3 seconds.

As a result of the elevated type I error rate exhibited by the resampling approach, we exclude it from the data analyses described in Section 3.2, Section 4, and in Section 1 of our Supplementary Materials. We retain ‘no aggregation’ in these analyses as a baseline for comparison.

### NSCLC application

We now compare the performance of the seven aggregation approaches on the NSCLC dataset described previously in Section 2.4 [28]. This dataset consisted of $153$ participants, each of whom had between four and six ROIs imaged. Each sample was labeled as exhibiting high or low major histocompatibility complex II (MHCII). A sample was labeled ‘high’ if greater than 5% of lung cancer cells expressed MHCII.

We treated MHCII-high status as a binary outcome and tested a specific set of hypotheses generated by the original study. Johnson *et al*. found that in tumors labeled MHCII-high, immune cells were closer to tumor cells than in MHCII-low samples. They also found that CD4 T cells and CD8 T cells more frequently colocalized with tumor cells in MHCII-high samples. We tested these three hypotheses using seven of the eight aggregation methods considered in our simulation study to assess which approach provided the most significance. Following the original analysis, we adjusted for patient age.

For testing, we used the SPatial Omnibus Test (SPOT) [15] to avoid having to select a radius at which to quantify the spatial summary. Instead, this test computes a spatial summary statistic, e.g. Ripley’s K, at a sequence of radii for each image. The association between the spatial summary and outcome is tested at each radius, yielding a sequence of $P$-values that are combined using the Cauchy combination test to yield an overall omnibus $P$-value. For each radius and each sample, we aggregated the spatial summaries across ROIs using an arithmetic mean, each of three weighted means, and two of the three ensemble approaches. We also considered treating each ROI independently for illustration. We used Ripley’s rule (the default in spatstat [29]) to generate a sequence of radii to consider. We considered 100 radii between $0$ and $123.625$.

The results are shown in Table 1. Across all hypotheses, the Baddeley mean provided the smallest $P$-values. This may be because there was not considerable variation in intensity across ROIs for tumor-immune cells, tumor-CD4 T cells, and tumor-CD8 T cells. The $P$-values for the Diggle mean and combo approach were similar in value and, in turn, suggested similar interpretations of the results (i.e., there may be significant associations between tumor-CD4 T cell colocalizations and MHCII-high TME. Note that these $P$-values are not adjusted for multiple testing and are intended to illustrate the variety of results each method provides.

**Table 1:** SPOT $P$-values testing the association between bivariate cell type colocalizations and MHCII-high status. An asterisk by ‘No Aggregation’ indicates this method is not a recommended approach to handling multiple spatial summary statistics. Note that these $P$-values are not adjusted for multiple testing.

SPOT P-Value			
Mean	Tumor-Immune	Tumor-CD4 T cell	Tumor-CD8 T cell
No Aggregation*	0.1842	0.0040	0.2209
Mean	0.4017	0.0808	0.3435
Baddeley	0.0698	0.0320	0.0403
Diggle	0.1302	0.0413	0.0576
Landau	0.2009	0.0948	0.0582
Ensemble	0.4155	0.0648	0.2288
Combo	0.1295	0.0347	0.0621

## Colorectal cancer application

We next compared the performance of the aggregation approaches in analyzing codetection by imaging (CODEX) of advanced-stage CRC tissue samples [30]. The dataset consisted of $140$ images collected from $35$ participants. Each sample was labeled as either exhibiting ‘Crohn’s-like reaction’ (CLR) ($n=18$) or ‘diffuse inflammatory infiltration’ (DII) ($n=17$). We treated the CRC type (CLR vs. DII) as a binary outcome with CLR as the reference level. We considered a range of radii between $0$ and $100$ with a step size of $10$ in accordance with previous analyses of this dataset [18]. We considered the spatial distribution of pairs of immune cells and tested their association with the log-odds of DII. We took a hypothesis-generating approach and tested all cell type pairs using the SPOT framework.

**Figure 4:**
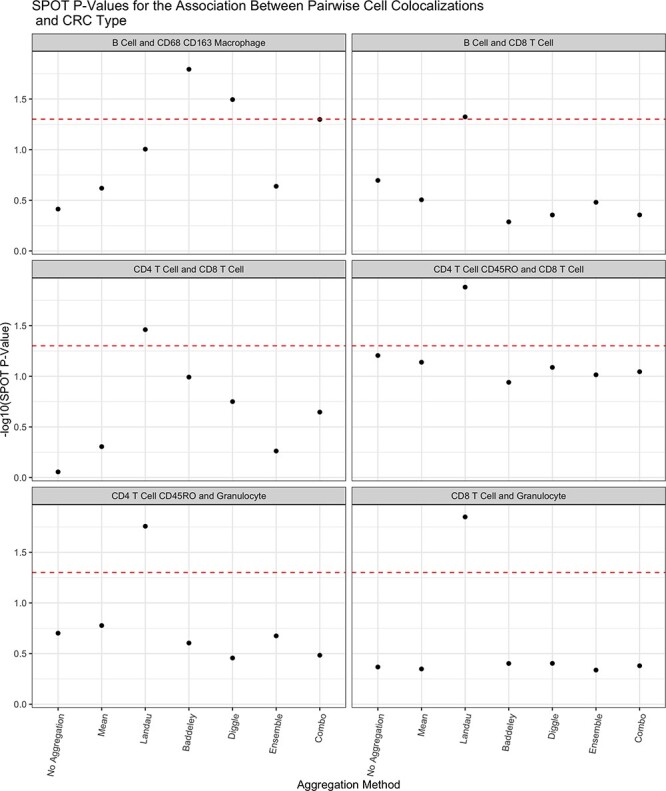
$P$ -values for the association between the Ripley’s K as a measure of spatial colocalization between immune cells and log-odds of a sample exhibiting diffuse inflammatory infiltration CRC. The red dashed line corresponds to a $P$-value of $0.05$ and $P$-values above this line are deemed significant. Note that these $P$-values have not been adjusted for multiple testing.

The results are shown in Fig. 4 for only cell type pairs with at least one significant $P$-value. Across most cell type pairs, the Landau mean provided the smallest $P$-values, suggesting a gain in power as a result of accounting for varying intensity across images. For the B cell–CD68 CD163 macrophage pair, the Baddeley, Diggle, and combo aggregators also showed significance.

## Discussion

The goal of our article was to examine the power and validity of aggregating spatial summary statistics across multiple ROIs using weighted means and ensemble testing in multiplexed spatial proteomics studies. In these studies, it is common for multiple regions of the tissue to be imaged to provide a more complete picture of the tumor microenvironment. A challenge is how to accommodate multiple spatial summary statistics estimated for the same sample. Standard weighted aggregations weight each spatial summary statistic based on either the number of cells or the estimated spatial point process intensity of each image. These are straightforward to implement and have a low computational burden. Ensemble tests, on the other hand, repeatedly generate many random weights and test for an association with the outcome using each of the resulting random means. This approach may better recover the optimal set of weights that reflects the true relationship between the spatial summary statistics and clinical outcomes. A drawback, however, is that these ensemble approaches require more computational time.

Overall, we found that ensemble testing performed no better in terms of power and validity than standard weighted averages, with the exception of resampling which had elevated type I error. Across the methods, we found if the intensity of the image is constant across images or is non-informative, using an arithmetic mean, the Diggle mean [21], the Baddeley mean [22], an ensemble testing approach, or a combination approach all offered high power and controlled type I error in simulations. If the intensity of each ROI varied or was informative for estimating the overall spatial summary statistic, the Landau mean [23] performed best. In practice, the Baddeley and Landau means offered the lowest $P$-values in our analysis of a non-small cell lung cancer study and of a CRC study. The Diggle mean and the combination ensemble test showed similar $P$-values and similar interpretations, highlighting their similarities.

Aggregating spatial summary statistics estimated from multiple ROIs of the same sample is straightforward. It allows us to obtain a single summary value for the spatial distribution of cells in each sample. We can treat this individual summary statistic as a covariate in any regression or outcome model suitable to our research questions. Another class of method that we did not examine include methods using random effects to account for intra-sample heterogeneity [18, 19]. These approaches treat the spatial summary statistic as the outcome and incorporate the clinical outcome as a covariate (e.g. treatment arm), but are difficult to generalize to survival outcomes. One could reverse the model and treat the clinical outcome as the dependent variable, but accommodating each type of clinical outcome would require a specially tailored random effects model with strong assumptions, e.g. on the mixing distribution, which could be far from the truth. Accordingly, this class of approach remains outside the scope of the present work.

In terms of selecting an approach to handling multiple ROIs, we found that the general ensemble and combination approaches performed comparably to the weighted means. Therefore, the choice may come down to computational burden: the ensemble approaches require more computing time than the weighted means. If opting for an ensemble approach, the number of ensemble replications will depend on the number of images in the study. More images would require more ensemble replications. On the other hand, among the weighted mean options an arithmetic mean performed no worse than the weighted means considered, though it is reasonable to consider a weighted mean that accounts for the number of cells in each image. With fewer cells, the spatial summary statistic may be less precise. As observed in our data applications, the Baddeley mean outperformed the Diggle and Landau means in the NSCLC application. In the CRC application, the Landau mean performed best, suggesting that the intensity of each image was an important factor in relating the spatial distribution of cells to the odds of DII-type CRC. In some cases, all methods provide the same results, as shown in the triple negative breast cancer application (Supplementary Materials).

We focused on the operating characteristics of using weighted means and ensemble testing approaches when describing the spatial qualities of cells using Ripley’s K [12]. However, we did not consider aggregating other spatial summary statistics, like the g-function [14], used for categorical marks, nor spatial summaries used for continuous marks like Moran’s I [31]. We also did not consider permutation-based approaches for handling gaps or tears in the tissue [10]. This is a common challenge in spatial proteomics imaging and violates the assumptions of homogeneity underlying popular spatial summary statistics, like Ripley’s K. To handle these situations, permuting the cell type labels to construct a null distribution and comparing the observed spatial summary statistic against this distribution is a suitable approach [32]. Aggregation within this permutation testing framework could be done to ensure robustness against deviations from homogeneity due to gaps in the images.

**Key Points**

- In multiplexed spatial proteomics studies, several disparate regions of the same tissue are often imaged to comprehensively describe the spatial arrangement of cells in a sample.
- We examined the operating characteristics of weighted aggregation methods and novel ensemble approaches for combining multiple spatial summary statistics estimated from the same sample when testing for an association with a sample-level endpoint.
- We found that a weighted average of the spatial summary statistics based on the number of cells in each image is a reasonable strategy for accommodating multiple spatial summaries that offers high power and effectively controls type I error.
- When the spatial intensity across images within the same sample varies, incorporating this information into the weighted aggregation of spatial summaries may yield additional power while still controlling type I error.
- A resampling approach, when a random image is selected from each tissue sample to use for association testing and the resulting $P$-values are combined across ensemble replications, is not recommended because it does not properly control type I error.

## Supplementary Material

Multiple_Images_Supplement_R1_bbae522

Multiple_Images_Supplement_R2_bbae522

## Data Availability

The data for the non-small cell lung cancer dataset was retrieved from http://juliawrobel.com/MI_tutorial/MI_Data.html. The data for the colorectal cancer application was retrieved from https://github.com/sealx017/SpaceANOVA/blob/main/Data/MIF_CRC.rda. The data for the triple negative breast cancer application was retrieved from https://zenodo.org/records/7990870.

## Software

An R package implementing these approaches is provided at https://sarahsamorodnitsky.github.io/spagg/index.html.
