# Supplementary material for: Statistical analysis of multiple regions-of-interest in multiplexed spatial proteomics data
Source: Brief Bioinform. 2024 Oct 20;25(6):bbae522. doi: 10.1093/bib/bbae522 (PMC11491162; doi:10.1093/bib/bbae522)
Supplement: Multiple_Images_Supplement_R1_bbae522 [file multiple_images_supplement_r1_bbae522.pdf]

Statistical Analysis of Multiple Regions-of-Interest in Multiplexed Spatial Proteomics Data: Supplementary Materials

Sarah Samorodnitsky[1,2], Michael C. Wu[1,2]

[1] Public Health Sciences Division, Fred Hutch Cancer Center

[2] SWOG Statistics and Data Management Center

1 Triple Negative Breast Cancer Application

We now consider applying different aggregation approaches to an analysis of triple negative breast cancer (TNBC). The data arises from an imaging mass cytometry experiment performed using TNBC core biopsies obtained from the NeoTRIP clinical trial [1]. NeoTRIP was a study comparing neoadjuvant chemotherapy against cheotherapy plus anti-PD-L1 immunotherapy. Samples were collected before, during, and after treatment. In this analysis, we examine the images obtained after treatment. Participants were categorized as either exhibiting pathological complete response (pCR) or recurrent disease (RD), which we treated as a binary outcome in our analysis.

Our analysis consisted of 210 participants with between one and three images collected post-treatment. Of these participants, 111 exhibited pCR while 99 experienced recurrence. Our analysis included 20 different cell types and we examine whether the spatial distribution of each cell type (as characterized by Ripley's K) is predictive of pCR. Using Ripley's rule and the SPOT method [2], we considered 100 radii between 0 and 72 as determined by Ripley's rule to consider a range

of candidate radii. At each radius, we use each of our aggregation methods, excluding resampling, and examine which approach yields the most significance.

The resulting p-values for each method and cell type are shown in Figure 1. The only cell type to show significant associations with pCR was PDPN+ stromal cells. Across all methods, the resulting p-values were significant and ranging in value from 0.0149 (Diggle's method) to 0.0197 (Landau's method). The remaining cell types did not show significance at the 0.05 level. Across hypotheses, all methods were in agreement regarding significance of the association between cell spatial distribution and pCR.

References

[1] Xiao Qian Wang, Esther Danenberg, Chiun-Sheng Huang, Daniel Egle, Maurizio Callari, Begoña Bermejo, Matteo Dugo, Claudio Zamagni, Marc Thill, Anton Anton, et al. Spatial predictors of immunotherapy response in triple-negative breast cancer. *Nature*, 621(7980):868–876, 2023.

[2] Sarah N Samorodnitsky, Katie M Campbell, Antoni Ribas, and Michael C Wu. A spatial omnibus test (spot) for spatial proteomic data. *Bioinformatics*, 40, 2024.

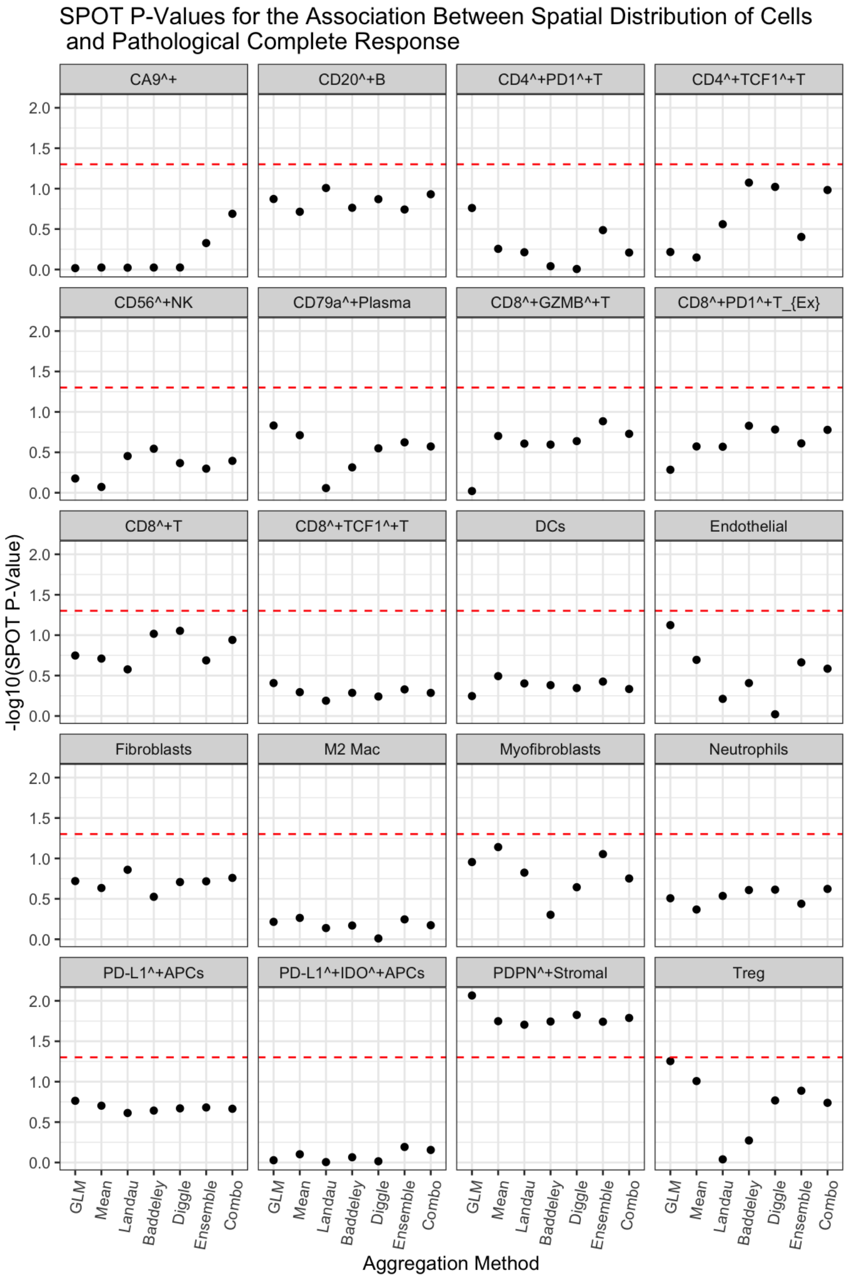

Figure 1: P-values characterizing the association between the spatial distribution of 20 immune cell types and pathologic complete response among TNBC patients.
